# Conventional imaging-derived phenotypes of brain abscess and short-term clinical outcomes: a comparative study in children and adults

**DOI:** 10.3389/fneur.2026.1810736

**Published:** 2026-08-07

**Authors:** Junxiu Guo, Bingliang Li, Wang Miao, Zhixiong Lin, Wen Jin, Hongxia Ren, Hongwei Xi, Zhaohe Liu

**Affiliations:** 1 Department of Neurosurgery, Shanxi Provincial Children’s Hospital, Taiyuan, China; 2 Department of Neonatal Surgery, Shanxi Provincial Children’s Hospital, Taiyuan, China; 3 Department of Pediatrics, Shanxi Medical University, Taiyuan, China; 4 Department of Neurosurgery, First Hospital of Shanxi Medical University, Taiyuan, China; 5 Department of Neurosurgery, Sanbo Brain Hospital, Capital Medical University, Beijing, China; 6 Department of General Surgery, Shanxi Provincial Children’s Hospital, Taiyuan, China

**Keywords:** brain abscess, conventional imaging features, imaging phenotype, phenotypic burden, risk stratification

## Abstract

**Objectives:**

To develop a composite imaging phenotype framework for brain abscess (BA) based on conventional imaging features, enabling systematic assessment of imaging heterogeneity, and to explore the distribution of these phenotypes across different age groups and their association with short-term clinical outcomes.

**Methods:**

This retrospective study consecutively enrolled patients with surgically confirmed BA admitted to two hospitals between January 2019 and December 2025. Patients were stratified into pediatric and adult groups based on age. All patients underwent surgical treatment and standard anti-infective therapy. Based on preoperative conventional imaging, three-dimensional composite phenotypes were defined: structural complexity, spatial complexity, and aggressive features. These were integrated into an “imaging phenotypic burden” score. Analyses examined age-related differences in phenotype distribution, associations with pathogen profiles, and the relationship between phenotypic burden and in-hospital adverse outcomes.

**Results:**

Significant differences were observed in imaging phenotype distribution between children and adults. Structural complexity was more common in the pediatric group (40.0% vs. 9.8%, *p* = 0.008), while spatial complexity was higher in adults (58.5% vs. 15.0%, *p* < 0.001). Microbiological analysis indicated a predominance of Streptococcus infections in children (*p* = 0.002); however, no stable statistical association was found between pathogen category and any specific imaging phenotype. A significant dose–response relationship existed between phenotypic burden and adverse outcome rates (*p* = 0.004, trend test). The high-burden group (≥2 phenotypes) had a significantly higher adverse outcome rate compared to the low-burden group (30.0% vs. 6.5%, *p* = 0.017). Deep-seated lesions were the primary anatomical factor driving high phenotypic burden and spatial complexity.

**Conclusion:**

The developed composite imaging phenotype framework effectively integrates information from conventional imaging to systematically assess BA heterogeneity. The imaging phenotypic burden is independently associated with short-term adverse outcomes. It serves as an intuitive, quantitative tool for early risk stratification, informing surgical timing decisions, and guiding intensive care resource allocation, demonstrating potential for clinical translation.

## 1 Introduction

Brain abscess (BA) remains one of the most severe focal infectious diseases of the central nervous system, characterized by persistently high mortality rates and a significant incidence of long-term neurological sequelae, posing ongoing challenges for clinical management (1, 2). This condition is relatively rare in children, often presenting with atypical clinical manifestations and a low frequency of the classic triad, which can lead to diagnostic delays (3, 4). Differences also exist between children and adults regarding the source of infection and causative pathogens. In children, direct extension from a contiguous focus is common, whereas adults typically exhibit a more complex microbiological spectrum (5, 6). Neuroimaging plays a pivotal role in the diagnosis and evaluation of BA. Contrast-enhanced magnetic resonance imaging (MRI), particularly in conjunction with diffusion-weighted imaging (DWI), has become the cornerstone diagnostic tool (7). Although the current mainstay strategy—combining stereotactic aspiration with prolonged antibiotic therapy—has improved outcomes, mortality remains high if severe complications such as intraventricular rupture occur. Approximately 30% of survivors are left with varying degrees of neurological deficits (8–10).

However, prior studies have primarily focused on correlating specific pathogens with imaging findings or have relied on advanced sequences like DWI for assessment (11, 12). Traditional approaches, dependent on interpreting isolated imaging signs, struggle to systematically quantify lesion heterogeneity across different dimensions, limiting their value for early risk stratification (13). Although conventional MRI and computed tomography (CT) sequences are widely used in clinical practice, the information they provide is often interpreted in isolation, lacking a standardized framework to integrate multi-dimensional features. Therefore, developing a method to systematically integrate conventional imaging features into an objective, comprehensive analytical tool for assessing BA heterogeneity represents a crucial direction for optimizing clinical decision-making. This study aims to construct a composite imaging phenotype analysis framework. By structurally integrating conventional MRI/CT features, it seeks to systematically describe imaging heterogeneity and investigate the distribution of these phenotypes in children versus adults, as well as their association with short-term clinical outcomes. The goal is to provide a new, practical perspective for early risk assessment.

## 2 Materials and methods

### 2.1 Study design and population

This retrospective study enrolled consecutive patients with surgically treated BA from the Department of Neurosurgery at Shanxi Children’s Hospital (age ≤18 years) and the First Hospital of Shanxi Medical University (age >18 years) between January 2019 and December 2025. The study protocol was approved by the Institutional Ethics Committee, with a waiver of informed consent. Inclusion criteria were: (1) Preoperative contrast-enhanced head MRI or CT showing typical features of BA (ring enhancement with central necrosis and surrounding edema); (2) Availability of complete preoperative clinical and imaging data; (3) Surgical confirmation of the diagnosis. Exclusion criteria included: (1) Death prior to or during hospitalization before surgery; (2) Missing or poor-quality imaging data; (3) Final diagnosis of a non-infectious lesion.

### 2.2 Treatment context

All patients received comprehensive treatment centered on surgical drainage. Indications for surgery included: abscess diameter ≥2.5 cm, significant mass effect, neurological deterioration, or failure of initial medical therapy (14). Perioperative empirical antimicrobial therapy was administered to all patients, with initial regimens covering common pathogens (e.g., vancomycin plus meropenem, or a third-generation cephalosporin plus metronidazole) (15–17). Treatment was adjusted postoperatively based on pus culture and susceptibility results. This standardized treatment approach provided a uniform therapeutic background for analyzing the association between imaging features and outcomes.

### 2.3 Data collection and variable definitions

Data were extracted from the electronic medical record system and included: (1) Baseline characteristics: age, sex, body mass index (BMI), clinical presentation (fever, impaired consciousness, intracranial hypertension, focal neurological deficits), and source of infection; (2) Imaging and laboratory parameters: imaging features (lesion location, number, size, degree of edema, mass effect), laboratory values (white blood cell count (WBC), procalcitonin (PCT) level), and microbiological data (intraoperative pus culture results categorized by genus, infection type, and clinical source); (3) Outcome measures: The primary outcome was an in-hospital adverse clinical outcome (death or new/persistent focal neurological deficit at discharge).

To analyze the association between imaging phenotypes and infection characteristics, some variables were combined for analysis: Infection source was categorized as “hematogenous” vs. “non-hematogenous” (e.g., direct spread from otogenic or sinus sources). Within the pediatric group, microbiology was categorized as “Streptococcus” vs. “Non-Streptococcus” (including other pathogens and culture-negative cases) to explore whether this most common pediatric pathogen (3) exhibited a specific imaging pattern. Furthermore, “deep-seated lesion” and “multiple lesions” were included as core anatomical variables for spatial complexity. Microbiological data served as exploratory variables for ancillary analysis.

### 2.4 Imaging evaluation and composite imaging phenotype definitions

All patients underwent preoperative conventional head MRI or CT (non-contrast and contrast-enhanced). Imaging assessments were performed independently and blindly by two physicians, one from radiology and one from neurosurgery, each with at least 5 years of experience. Inter-observer agreement was assessed using Kappa statistics, which showed good to excellent consistency for all three phenotype dimensions: structural complexity *κ* = 0.817 (95% CI: 0.684–0.936), aggressive features κ = 0.779 (95% CI: 0.651–0.892), and spatial complexity κ = 0.852 (95% CI: 0.723–0.958), with an overall agreement of κ = 0.808 (95% CI: 0.702–0.913). For cases with discrepant assessments, consensus was reached through discussion. If disagreement persisted, a third senior imaging specialist arbitrated.

The composite imaging phenotype framework constructed in this study comprised three dimensions, with specific imaging definitions and constituent features as follows:

Structural Complexity: Refers to an irregular abscess capsule morphology, heterogeneous enhancement, or internal septations (18). Specifically includes: (1) Incomplete capsule or heterogeneous enhancement; (2) Internal compartmentalization (septations).

Aggressive Features: Refers to lesions causing significant mass effect or invasion of adjacent structures (19, 20). Mainly includes: (1) Moderate-to-severe perilesional edema (see quantitative criteria below); (2) Midline shift ≥5 mm; (3) Ventricular wall involvement or intraventricular rupture.

Spatial Complexity: Refers to lesions located in deep functional areas or presenting as multiple foci (2, 21). Specifically manifested as: (1) Deep location (e.g., basal ganglia, thalamus, brainstem); (2) Multiple lesions (≥2).

The determination of “Moderate-to-severe edema” employed quantitative criteria: On axial T2/FLAIR sequences, the widest perpendicular distance of the edema band from the abscess wall was measured. Mild edema was defined as width ≤1 cm or confined to a perilesional halo. Moderate edema was defined as width 1–2 cm and/or confined to the same lobe, with mild-to-moderate mass effect. Severe edema was defined as width >2 cm and/or extending across lobes, involving white matter tracts, or presenting as confluent patchy edema with significant mass effect.

To integrate this multi-dimensional information, “Imaging Phenotypic Burden” was defined as the number of the above phenotypes a patient simultaneously met (range 0–3), serving as a semi-quantitative indicator of overall lesion aggressiveness.

### 2.5 Outcome measures

The primary outcome was an adverse clinical outcome, defined as death during hospitalization or new/persistent focal neurological deficit (e.g., hemiparesis, aphasia, visual field defect) at discharge. Due to the retrospective nature of this study, quantitative neurological scales (e.g., GCS and mRS) were not systematically documented; therefore, we used the binary indicator of new or persistent focal neurological deficit at discharge, which was reliably extracted from discharge summaries. This binary measure reflects both acute disease severity and initial treatment efficacy.

### 2.6 Statistical analysis

Statistical analysis was performed using SPSS 26.0 software. Continuous variables following a normal distribution are presented as mean ± standard deviation and compared using the independent samples *t*-test. Non-normally distributed continuous variables are presented as median (interquartile range, IQR) and compared using the Mann–Whitney *U* test. Categorical variables are presented as number (percentage) and compared using the Chi-square test or Fisher’s exact test. When any expected cell count was <5, Fisher’s exact test was used. For Fisher’s exact test, no test statistic is reported and “-” is used in tables. The dose–response relationship between phenotypic burden and adverse outcome was assessed using the Cochran-Armitage trend test. For risk stratification, patients were divided into a Low-Burden group (0–1 phenotypes) and a High-Burden group (≥2 phenotypes), with adverse outcome rates compared using the Chi-square test. The relationship between microbiology and imaging phenotypes was analyzed by age stratification: within the pediatric group, the most common bacterial species was compared; within the adult group, a descriptive analysis was performed. To further explore the determinants of phenotype distribution beyond age, we conducted an additional exploratory analysis across the entire cohort, examining the associations of key anatomical factors (deep-seated lesion, multiple lesions) and infection route with each phenotype and high phenotypic burden, to test whether anatomical variables exert an independent effect on imaging phenotypes irrespective of age. All tests were two-sided, with *p* < 0.05 considered statistically significant.

## 3 Results

### 3.1 Baseline characteristics of the study population

A total of 61 patients with surgically confirmed BA were enrolled, including 20 in the pediatric group (≤18 years) and 41 in the adult group. A comparison of baseline characteristics between the two groups is presented in Table 1. Apart from expected differences in age and body mass index (BMI) between the pediatric and adult groups, no statistically significant differences were observed in gender or the incidence of fever, impaired consciousness, intracranial hypertension, and focal neurological deficits. The distribution of infection sources showed significant heterogeneity between the pediatric and adult groups (*p* < 0.001). Laboratory tests revealed higher WBC and PCT levels in the pediatric group compared to the adult group. Radiologically, the adult group had significantly higher proportions of deep-seated lesions, supratentorial deep lesions, and multiple lesions compared to the pediatric group. The maximum abscess diameter was smaller in adults than in the pediatric group, but no differences were found in estimated lesion volume, ventricular invasion, or lesion laterality between the groups. Regarding the treatment course, the pediatric group had a shorter interval from symptom onset to hospital admission than the adult group, but a longer interval from admission to surgery, a larger volume of intraoperative pus, and a significantly longer total hospital stay compared to the adult group. The rates of intensive care unit (ICU) admission and the incidence of in-hospital adverse clinical outcomes (death or new neurological deficits) were similar between the pediatric and adult groups.

**Table 1:** Comparison of baseline characteristics between pediatric and adult patients with brain abscess.

Variable	Pediatric (*n* = 20)	Adult (*n* = 41)	Statistic	*p*
Age, years, Median (IQR)	3 (1, 10)	53 (40, 62)	6.302	<0.001
Male, *n* (%)	15 (75.0%)	28 (68.3%)	0.291	0.411
BMI, kg/m^2^, Median (IQR)	17.54 (14.88, 19.044)	25.25 (20.89, 27.61)	4.832	<0.001
Fever, *n* (%)	14 (70.0%)	20 (48.8%)	2.453	0.098
Impaired consciousness, *n* (%)	9 (45.0%)	14 (34.1%)	0.674	0.293
Intracranial hypertension, *n* (%)	14 (70.0%)	33 (80.5%)	0.836	0.274
Focal neurological deficit, *n* (%)	12 (60.0%)	19 (46.3%)	1.003	0.233
Source of infection, n (%)			—	<0.001
Sinonasal origin	9 (45.0%)	3 (7.3%)	—	<0.001
Otogenic origin	1 (5.0%)	7 (17.1%)	—	0.185
Hematogenous origin	9 (45.0%)	10 (24.4%)	—	0.092
Other/Unknown origin	1 (5.0%)	21 (51.2%)	—	<0.001
White blood cell count (×10^9^/L), Median (IQR)	12.95 (10.69, 17.00)	10.70 (8.70, 14.05)	2.435	0.015
Neutrophil percentage (%), Median (IQR)	73.0 (61.3, 84.7)	82.3 (70.8, 87.1)	0.638	0.524
Procalcitonin (ng/mL), Median (IQR)	0.734 (0.074, 3.210)	0.104 (0.023, 0.684)	2.076	0.038
Number of lesions, *n* (%)			4.726	0.027
Single lesion	18 (90.0%)	26 (63.4%)		
Multiple lesions	2 (10.0%)	15 (36.6%)		
Max. abscess diameter, cm, Mean ± SD	4.49 ± 1.57	4.07 ± 1.01	2.746	0.008
Estimated volume, cm^3^, Median (IQR)	31.3 (25.2, 54.9)	22.2 (16.9, 34.3)	1.506	0.132
Deep-seated lesion, *n* (%)	2 (10.0%)	20 (48.8%)	8.768	0.003
Ventricular invasion, *n* (%)	3 (15.0%)	3 (7.3%)	—	0.303
Lesion location, *n* (%)			—	0.005
Supratentorial, superficial	20 (100%)	25 (61.0%)	—	0.001
Supratentorial, deep	0 (0%)	12 (29.3%)	—	0.005
Infratentorial (cerebellar)	0 (0%)	4 (9.8%)	—	0.194
Laterality, *n* (%)			—	0.370
Left	14 (70.0%)	22 (53.7%)	—	0.174
Right	5 (25.0%)	18 (43.9%)	—	0.125
Bilateral	1 (5.0%)	1 (2.4%)	—	0.552
Onset-to-admission interval, days, Median (IQR)	3 (2, 10)	15 (7, 26)	2.671	0.008
Admission-to-surgery interval, days, Median (IQR)	7 (2, 13)	3 (2, 5)	2.226	0.026
Intraoperative pus volume, ml, Median (IQR)	25 (20, 35)	15 (10, 25)	1.961	0.049
Hospital stay, days, Median (IQR)	42 (33, 45)	23 (15, 31)	3.782	<0.001
ICU admission, *n* (%)	1 (5.0%)	3 (7.3%)	—	0.603
Adverse clinical outcome, *n* (%)	3 (15.0%)	8 (19.5%)	—	0.481

### 3.2 Overall distribution of composite imaging phenotypes and age-related differences

**Figure 1:**
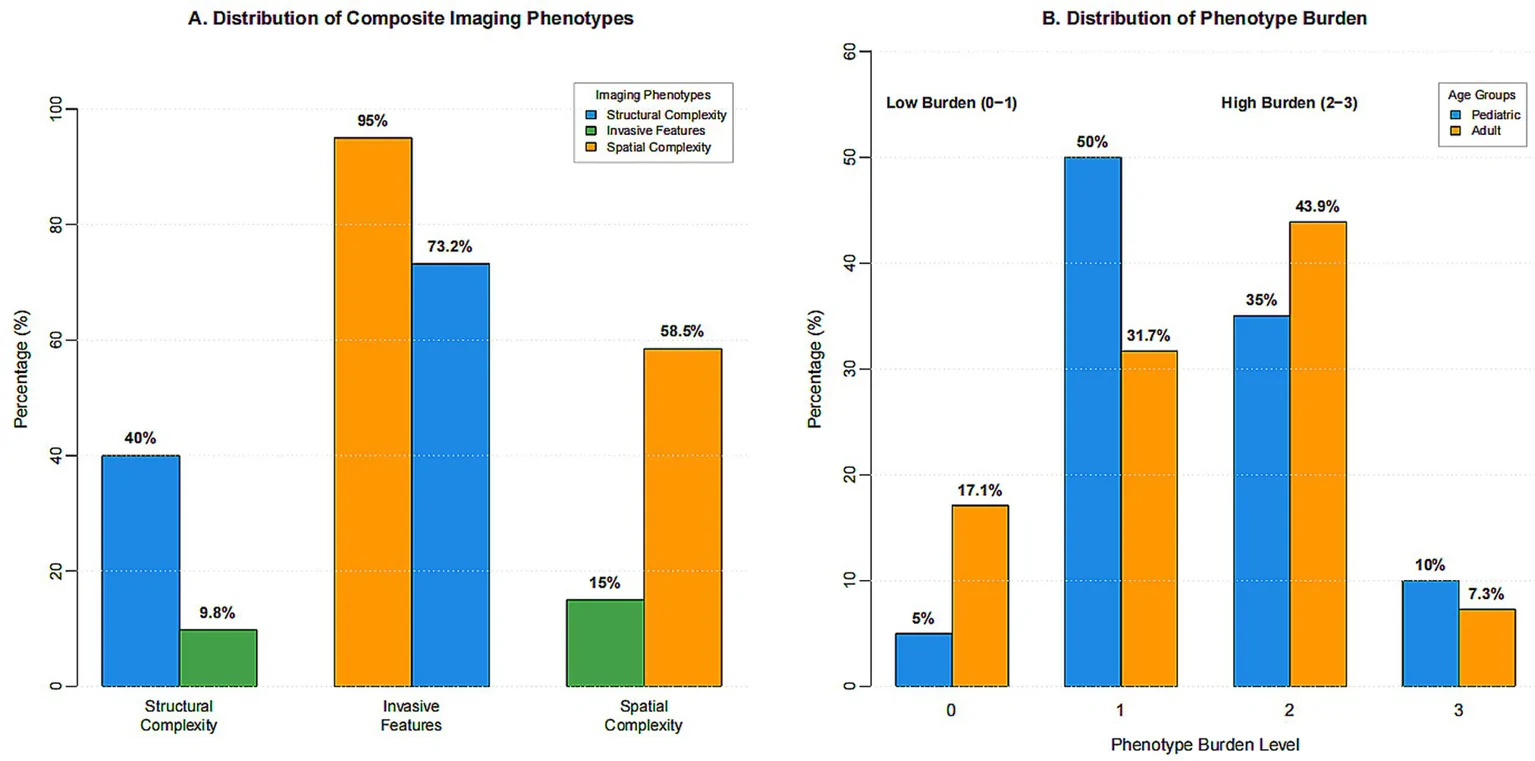
Distribution of composite imaging phenotypes and phenotypic burden in pediatric and adult brain abscess patients. Panel **(A)** shows percentages of the three phenotypes; Panel **(B)** shows burden level distribution.

**Figure 2:**
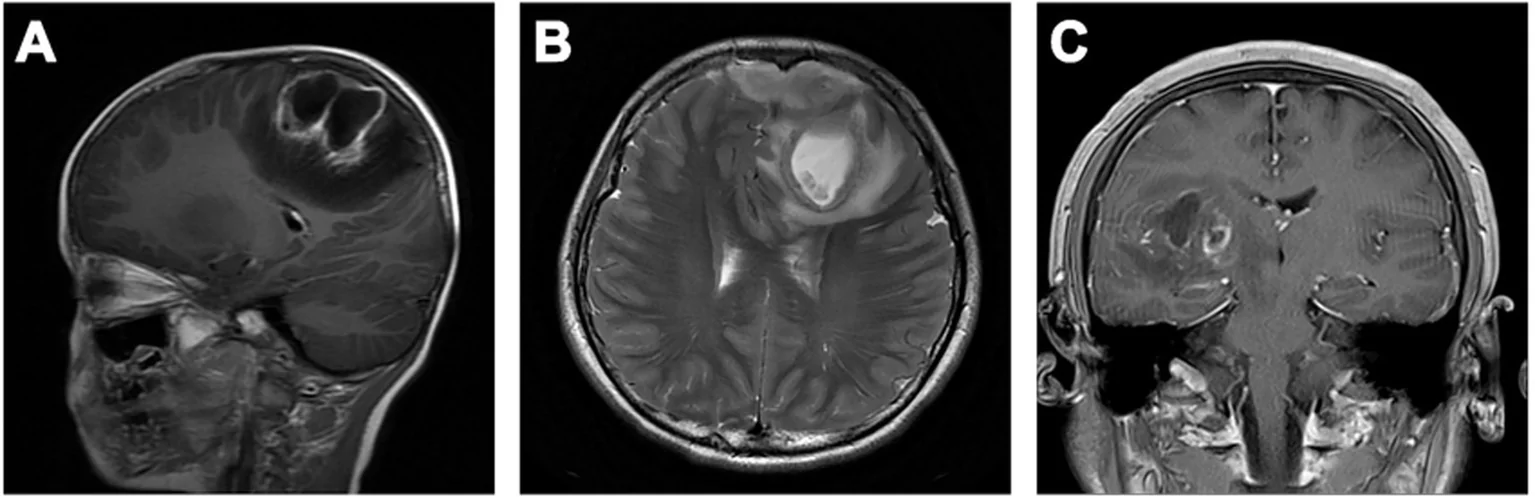
Typical imaging examples of brain abscess composite phenotypes (**A–C** from different patients). **(A)** Contrast-enhanced T1-weighted MRI shows an irregular, septated lesion with rim enhancement in the right basal ganglia (structural + spatial complexity). **(B)** Axial T2/FLAIR image demonstrates a left frontal abscess with extensive perilesional edema and midline shift (aggressive features). **(C)** Contrast-enhanced T1-weighted MRI reveals multiple ring-enhancing lesions involving bilateral hemispheres, with one lesion located in the thalamus (spatial complexity).

The distribution of the three composite imaging phenotypes overall and across age groups is shown in Table 2. Overall, aggressive features were the most common, followed by spatial and structural complexity. Phenotype distribution differed significantly between children and adults: structural complexity was more common in children, while spatial complexity was higher in adults. Aggressive features were highly prevalent in both groups but more so in children. Although the distribution of phenotypic burden did not differ statistically between groups, pediatric patients were mostly concentrated in the low-burden category (0–1 phenotypes), whereas a higher proportion of adults fell into the high-burden category (2–3 phenotypes) (Figure 1). Figure 2 shows typical imaging examples of cases meeting two or more phenotypes: Panel A (contrast-enhanced T1-weighted MRI) shows an irregular rim-enhancing lesion in the right basal ganglia with internal septations and heterogeneous enhancement, representing structural complexity combined with spatial complexity due to its deep location; Panel B (axial T2/FLAIR) demonstrates a large left frontal abscess with extensive perilesional edema extending across lobes and a midline shift of 8 mm, meeting the criteria for aggressive features; and Panel C (contrast-enhanced T1-weighted MRI) reveals multiple ring-enhancing lesions in bilateral cerebral hemispheres, with one lesion located in the thalamus, illustrating spatial complexity with multifocality and deep involvement.

**Table 2:** Distribution of composite imaging phenotypes and phenotypic burden in children and adults.

Phenotype/Phenotypic burden	Pediatric (*n* = 20)	Adult (*n* = 41)	Statistic	*p*
Single phenotype, *n* (%)				
Structural complexity	8 (40.0%)	4 (9.8%)	—	0.008
Aggressive features	19 (95.0%)	30 (73.2%)	—	0.041
Spatial complexity	3 (15.0%)	24 (58.5%)	—	<0.001
Phenotypic burden, *n* (%)			—	0.096
0 phenotypes	1 (5.0%)	7 (17.1%)		
1 phenotype	10 (50.0%)	13 (31.7%)		
2 phenotypes	7 (35.0%)	18 (43.9%)		
3 phenotypes	2 (10.0%)	3 (7.3%)		

Categorical variables are presented as number (percentage). Between-group comparison of phenotypic burden distribution was also performed using Fisher’s exact test, as expected cell counts were <5 in some cells; for Fisher’s exact test, no test statistic is reported.

### 3.3 Association between imaging phenotypic burden and clinical outcomes

**Figure 3:**
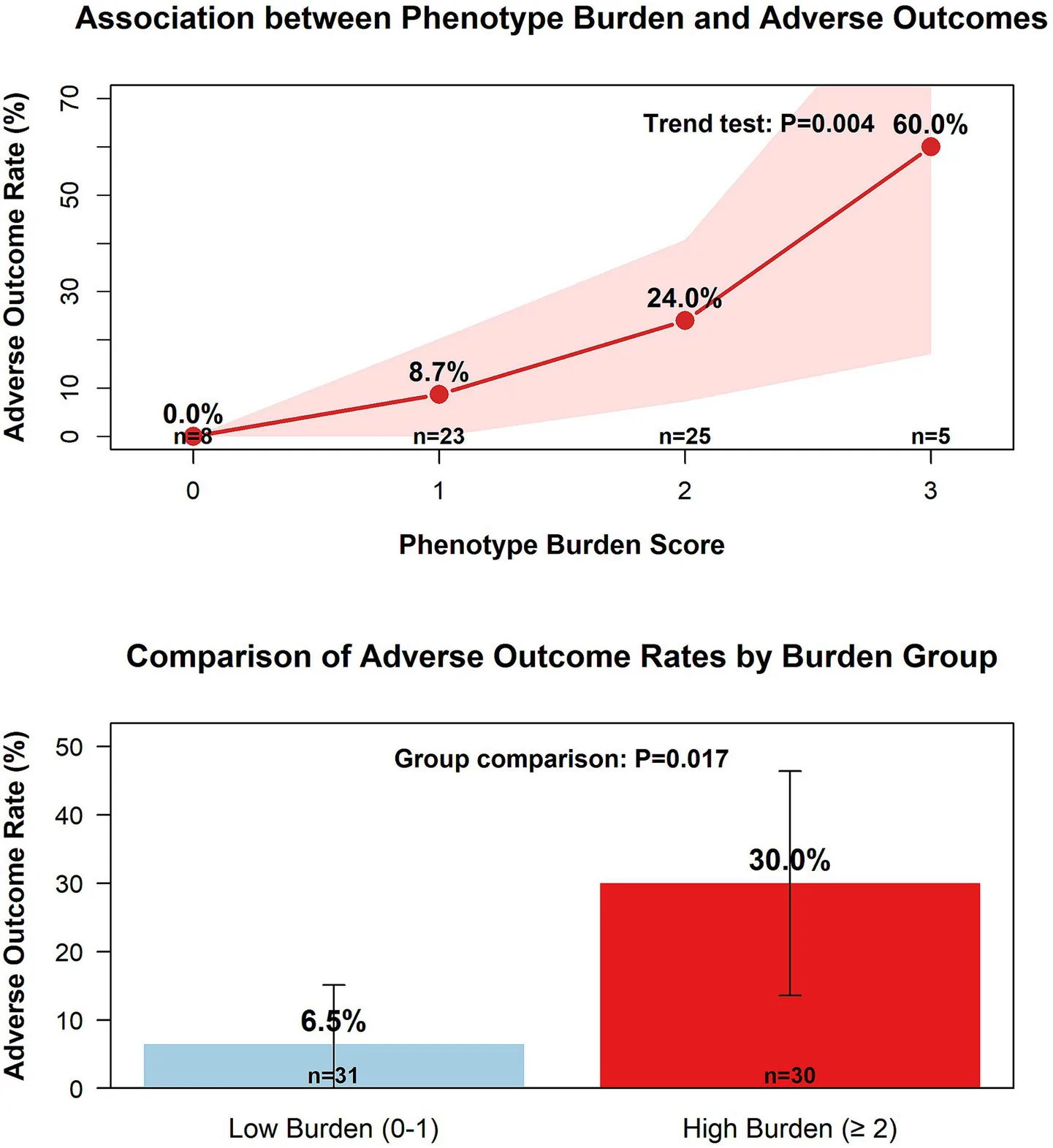
Association between imaging phenotypic burden and adverse clinical outcomes. The bar chart shows the comparison of adverse outcome rates between the low-burden group (0–1 phenotypes) and the high-burden group (≥2 phenotypes), with *p* = 0.017 (Chi-square test); the overlaid line graph illustrates the dose–response trend across four ordinal burden levels (0, 1, 2, 3 phenotypes), with *p* = 0.004 (trend test).

The analysis of the association between imaging phenotypic burden and adverse clinical outcomes is shown in Table 3 and Figure 3. The incidence of adverse outcomes progressively increased with higher levels of phenotypic burden, from 0% in the 0-phenotype group to 60.0% in the 3-phenotype group, demonstrating a significant dose–response relationship (*p* = 0.004, trend test). When dichotomized for clinical risk stratification, the high-burden group (≥2 phenotypes) had a significantly higher adverse outcome rate compared to the low-burden group (0–1 phenotypes) (30.0% vs. 6.5%, *p* = 0.017).

**Table 3:** Relationship between imaging phenotypic burden and adverse clinical outcomes.

Phenotypic burden	Total	Adverse outcome *n* (%)	Statistic	*p*
A. Trend analysis (by burden level)			8.385	0.004
0 phenotypes	8	0 (0.0%)		
0 phenotype	23	2 (8.7%)		
0 phenotypes	25	6 (24.0%)		
0 phenotypes	5	3 (60.0%)		
B. Group comparison			5.718	0.017
Low Burden (0–1 phenotypes)	31	2 (6.5%)		
High Burden (≥2 phenotypes)	30	9 (30.0%)		

Part A used the Cochran-Armitage trend test to assess if adverse outcome rates increased with higher phenotypic burden levels. Part B used the Chi-square test.

### 3.4 Anatomical and disease-related factors influencing composite phenotype distribution

To explore potential determinants of composite phenotype distribution, we analyzed the association of key anatomical factors (deep-seated lesion, multiple lesions) and infection route (hematogenous vs. non-hematogenous) with each phenotype based on the aforementioned definitions (Table 4). Lesion location was the primary factor influencing phenotype distribution. Deep-seated lesions showed significant positive correlations with both spatial complexity and high phenotypic burden. In contrast, the number of lesions was associated only with spatial complexity. The source of infection showed no significant association with any imaging phenotype or high phenotypic burden.

**Table 4:** Association analysis of key clinical and imaging features with composite imaging phenotypes.

Feature	Category	Structural complexity [*n* (%)]	Aggressive features [*n* (%)]	Spatial complexity [*n* (%)]	High phenotypic burden [*n* (%)]
Deep-seated lesion	Yes (*n* = 22)	4 (18.2%)	18 (81.8%)	22 (100.0%)	16 (72.7%)
	No (*n* = 39)	8 (20.5%)	31 (79.5%)	5 (12.8%)	14 (35.9%)
	*p*	1.000	1.000	<0.001	0.009
Multiple lesions	Yes (*n* = 17)	1 (5.9%)	15 (88.2%)	17 (100.0%)	12 (70.6%)
	No (*n* = 44)	11 (25.0%)	34 (77.3%)	10 (22.7%)	18 (40.9%)
	*p*	0.672	0.682	<0.001	0.042
Source of infection	Hematogenous (*n* = 19)	4 (21.1%)	15 (78.9%)	10 (52.6%)	10 (52.6%)
	Non-hematogenous (*n* = 42)	8 (19.0%)	34 (81.0%)	17 (40.5%)	20 (47.6%)
	*p*	1.000	1.000	0.387	0.733

Data are presented as number (percentage). Comparisons of incidence across feature categories for specific phenotypes used Fisher’s exact test. Total positive cases: Structural complexity *n* = 12, Aggressive features *n* = 49, Spatial complexity *n* = 27, High Phenotypic Burden (≥2 phenotypes) *n* = 30.

### 3.5 Microbiological distribution and exploratory analysis of its association with imaging phenotypes

Among all 61 patients, definitive microbiological results were obtained in 38 (62.3%). The pathogen spectrum differed significantly between children and adults (Supplementary Table 1). The pediatric group was dominated by Streptococcus species, with higher proportions of monomicrobial and community-acquired infections. In adults, there were higher proportions of Gram-negative bacilli, polymicrobial infections, and hospital-acquired/drug-resistant pathogens.

To explore the influence of pathogens on imaging appearance, stratified analysis was performed (Supplementary Table 2). Within the pediatric group, no statistical differences were found in the incidence of any imaging phenotype or high phenotypic burden between the most common Streptococcus infections and non-Streptococcus infections. In the adult group, no clear association pattern was observed between different pathogen categories and imaging phenotypes.

**Figure 4:**
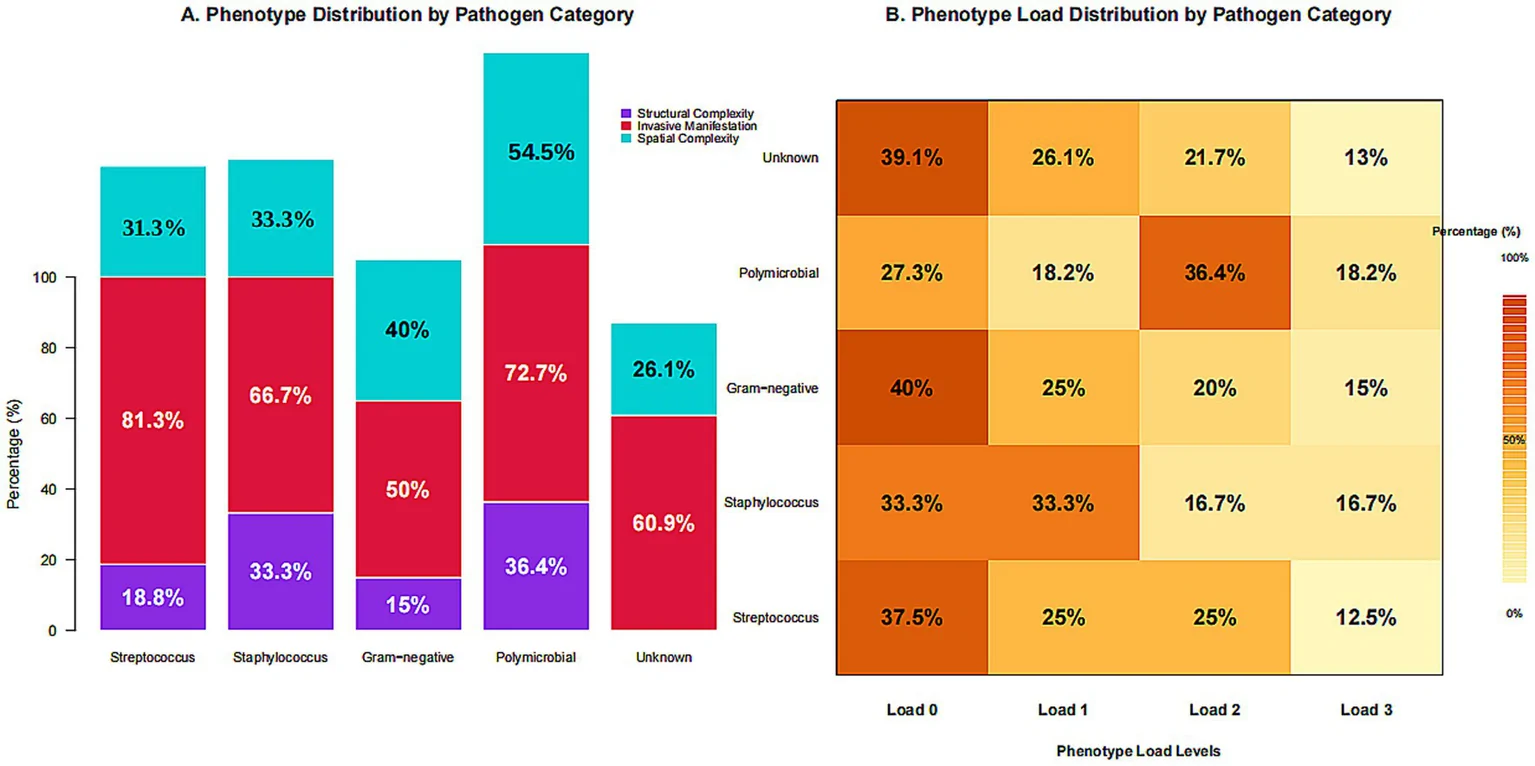
Distribution of imaging phenotypes across different microbiological classifications. Panel **(A)** is the bar chart by pathogen category; Panel **(B)** is the heatmap of burden levels.

In exploratory descriptions, some distribution trends were noted (Figure 4; Supplementary Table 3): Aggressive features were relatively common across all pathogen categories; Staphylococcus infections showed a higher proportion of structural complexity; Gram-negative bacilli and polymicrobial infection groups had relatively higher proportions of spatial complexity and high phenotypic burden. Furthermore, among patients with high phenotypic burden (≥2 phenotypes), specific co-occurrence patterns of phenotypes were observed, with “Aggressive features + Spatial complexity” being the most common (Supplementary Table 4). Comprehensive analysis suggests a lack of stable association between pathogen type and the composite imaging phenotypes defined in this study.

## 4 Discussion

Based on clinical data, this study developed and validated a composite phenotype system for assessing imaging heterogeneity in BA. Three key findings emerged: first, significant differences exist in imaging phenotype distribution between pediatric and adult patients; second, no stable association was found between pathogen type and imaging phenotype; third, a clear dose–response relationship exists between “imaging phenotypic burden” and the risk of in-hospital adverse outcomes. These results suggest that the imaging manifestations of BA likely reflect the host’s local and systemic pathophysiological response rather than direct features of specific pathogens.

This study aimed to address a common clinical need: whether a systematic and reproducible risk assessment of BA severity can be performed based on conventional contrast-enhanced MRI or CT images before microbiological diagnosis is confirmed. Traditional methods often rely on single imaging features, which offer limited informational dimensions and are susceptible to subjective interpretation (2, 13). To address this, we constructed a composite imaging phenotype framework encompassing three dimensions—structural complexity, spatial complexity, and aggressive features. This framework aims to integrate scattered features from conventional imaging into a structured assessment system, thereby more comprehensively characterizing the continuous pathological process of BA from the cerebritis stage to capsule formation (18). It is important to clarify that this framework is not intended to replace the role of advanced sequences like DWI in differential diagnosis and treatment response evaluation (7), but rather to complement them. Conventional sequences primarily reflect morphological and spatial features (e.g., capsule morphology, edema, mass effect), whereas DWI focuses more on physiological aspects like cytotoxic edema. By deconstructing and integrating conventional imaging information, our framework establishes an independent “structured imaging risk parsing tool.” This tool enables rapid, objective risk stratification while awaiting microbiological results. When combined with advanced imaging, it can provide a more comprehensive and refined assessment of disease aggressiveness, thereby offering multidimensional imaging evidence for clinical decision-making.

In the initial phase of this research, we attempted to establish a stable correlation between imaging findings and causative pathogens. However, the view that imaging manifestations serve as pathogen-specific markers lacks robustness at the population level. Both this study and prior data indicate that specific pathogen types are poor consistent predictors of imaging patterns (4). For instance, Streptococcus infections can present as either solitary, superficial, regular abscesses or as multiple, deep, or structurally complex lesions. This study found that although the pathogen spectrum in adults is typically more complex (including more hospital-acquired and drug-resistant strains), their imaging was characterized by increased “spatial complexity.” In contrast, pediatric patients, despite having a predominance of community-acquired pathogens, more frequently exhibited “structural complexity” and “aggressive features” on imaging. This indicates that imaging manifestations result from the combined effects of the pathogen, host immune response, local anatomical microenvironment, and disease stage.

We posit that these age-related imaging differences may stem from systematic variations in anatomy and physiology. In children, particularly infants, the blood–brain barrier is not fully developed, and brain tissue has higher water content. This may allow inflammatory responses to spread more easily and become less contained, manifesting on imaging as irregular capsule formation and significant vasogenic edema (22, 23). In contrast, deep gray matter nuclei in adults (e.g., basal ganglia) are primarily supplied by end-arteries with limited collateral circulation. This vascular anatomy may predispose hematogenously disseminated infectious emboli to lodge in these areas, leading more readily to the formation of deep or multiple abscesses, corresponding to a higher “spatial complexity” score (21). Factor analysis of phenotype distribution further confirmed that anatomical location is the primary determinant influencing imaging phenotypes, outweighing the effect of pathogen type. Nearly all deep-seated lesions met the criteria for the “spatial complexity” phenotype and predominantly belonged to the high phenotypic burden group. Even when categorized by infection route into “hematogenous” and “non-hematogenous,” neither showed a significant association with any imaging phenotype. This aligns with the anatomical reality that deep-seated lesions are adjacent to critical neural structures and the ventricular system, also explaining from an imaging perspective why they are more prone to cause severe neurological deficits or ventricular rupture (20, 21). Therefore, we interpret imaging phenotypes as comprehensive morphological manifestations arising from the interaction between an infection and a specific host (with age-dependent anatomical and microenvironmental contexts). They primarily reflect the overall pathological process of the “host-pathogen” interaction and the lesion’s “functional risk level” based on its anatomical location, rather than merely pointing to pathogen characteristics.

The “phenotypic burden” metric derived from our framework (i.e., the number of imaging phenotypes a patient meets simultaneously) holds clear clinical significance. This metric is simple to calculate and shows a significant incremental association with poor prognosis. Its pathophysiological basis lies in the premise that lesions simultaneously exhibiting deep location, structural disorganization, and significant mass effect suggest multiple injury mechanisms concurrently affecting the central nervous system, potentially exceeding the brain’s functional compensation threshold more easily (19, 24). Thus, phenotypic burden can be viewed as a continuous variable reflecting the degree of disease aggressiveness. From a clinical decision-making perspective, this metric provides an objective, immediate stratification basis for determining early treatment intensity and monitoring strategy. Before microbiological results are available, a high phenotypic burden (≥2) indicates a lesion with high-risk anatomical location, local structural disruption, and significant mass effect. Patients in this category are at higher risk of neurological deterioration, warranting more aggressive antimicrobial coverage, early surgical planning, and intensive care. Conversely, patients with low burden can, on the basis of clinical stability, afford more time for comprehensive microbiological evaluation and individualized treatment planning. It is crucial to emphasize that imaging phenotypic burden is not a substitute for clinical judgment. Rather, it serves as an imaging-based, reproducible, and interpretable risk assessment and communication tool for multidisciplinary teams during the information-limited phase.

The composite imaging phenotype framework developed in this study is constructed entirely based on features from conventional contrast-enhanced imaging (MRI/CT), offering good clinical accessibility and reproducibility. Its purpose is not as a precise predictive model but as an analytical tool for the structured parsing and standardized description of disease complexity. The resulting phenotype combinations and burden scores can provide an objective basis for determining treatment intensity, formulating follow-up plans, and facilitating doctor-patient communication. In the context of this study, where all patients received guideline-adherent surgical drainage and prolonged antimicrobial therapy (2, 8, 16), the association between phenotypic burden and adverse outcomes further highlights its value for early, independent risk assessment prior to treatment, suggesting that initial disease severity remains a key prognostic factor.

This study has several limitations that must be fully considered when interpreting the results. First, the retrospective design, limited sample size, and recruitment primarily from two institutions in the same region may restrict the generalizability of the conclusions. Second, phenotype assessment relied on manual interpretation; future validation through consistency checks or automated methods is needed. Third, standardized quantitative neurological scales (e.g., GCS or mRS) were not systematically documented, limiting our ability to perform graded neurological assessments; we therefore relied on binary clinical indicators for outcome evaluation. Fourth, CRP data were incomplete, precluding a systematic correlation analysis with other inflammatory biomarkers. Microbiological analyses were exploratory, with small sample sizes in some subgroups. The association between phenotypic burden and outcomes might be influenced by confounders like lesion location, which this study could not fully adjust for using multivariate models. The outcome measure was limited to short-term, in-hospital outcomes, lacking assessment of long-term neurological function and quality of life. Future large-scale, multicenter prospective studies are warranted to further validate and refine this composite imaging phenotype framework.

## 5 Conclusion

In conclusion, the composite imaging phenotype framework developed in this study effectively integrates conventional imaging features to systematically assess BA heterogeneity. It provides an intuitive, reproducible tool for early risk quantification, enabling preoperative risk stratification and offering an objective basis for clinical decision-making and resource allocation. This framework complements advanced imaging techniques, collectively enhancing the efficacy of BA diagnosis, treatment, and evaluation.

## Data Availability

The raw data supporting the conclusions of this article will be made available by the authors, without undue reservation.
