# Supplementary material for: Conventional imaging-derived phenotypes of brain abscess and short-term clinical outcomes: a comparative study in children and adults
Source: Front Neurol. 2026 Aug 7;17:1810736. doi: 10.3389/fneur.2026.1810736 (PMC13493290; doi:10.3389/fneur.2026.1810736)
Supplement: Supplementary file 1 [file Table_1.DOCX]

**Supplementary Table 1. Distribution of Pathogens in Pediatric and Adult Patients with Brain Abscess**

| **Grouping** | **Pathogen Category** | **Pediatric, n (%)** | **Adult, n (%)** | **Statistic** | **P** |
| --- | --- | --- | --- | --- | --- |
| **By Genus** | **Streptococcus spp.** | **11 (55.0%)** | **5 (12.2%)** | **9.426** | **0.002** |
|  | **Staphylococcus spp.** | **2 (10.0%)** | **4 (9.8%)** | **-** | **1.000** |
|  | **Gram-negative bacilli** | **4 (20.0%)** | **16 (39.0%)** | **2.065** | **0.151** |
|  | **Culture-negative/Unspecified** | **5 (25.0%)** | **18 (43.9%)** | **2.045** | **0.153** |
| **By Infection Type** | **Monomicrobial infection** | **13 (65.0%)** | **14 (34.1%)** | **4.946** | **0.026** |
|  | **Polymicrobial infection** | **2 (10.0%)** | **9 (22.0%)** | **-** | **0.482** |
|  | **Culture-negative/Unspecified** | **5 (25.0%)** | **18 (43.9%)** | **2.045** | **0.153** |
| **By Clinical Feature** | **Community-acquired/common pathogens** | **13 (65.0%)** | **7 (17.1%)** | **14.004** | **<0.001** |
|  | **Hospital-acquired/Drug-resistant pathogens** | **2 (10.0%)** | **16 (39.0%)** | **5.444** | **0.020** |
|  | **Culture-negative/Unspecified** | **5 (25.0%)** | **18 (43.9%)** | **2.045** | **0.153** |

Note: Data are presented as number (percentage). Pathogens from mixed infections were split and counted individually in the corresponding genus category. Between-group comparisons were performed using Fisher's exact test.

**Supplementary Table 2. Distribution of Imaging Phenotypes by Pathogen Category within Age Strata**

| **Grouping / Phenotype** | **Streptococcus [n (%)]** | **Non-Streptococcus [n (%)]** | **Statistic** | **P** |
| --- | --- | --- | --- | --- |
| **A. Pediatric Patients (n=20)** | **n=11** | **n=9** |  |  |
| **Structural complexity** | **4 (36.4%)** | **4 (44.4%)** | **-** | **0.429** |
| **Aggressive features** | **11 (100.0%)** | **8 (88.9%)** | **-** | **0.569** |
| **Spatial complexity** | **1 (9.1%)** | **2 (22.2%)** | **-** | **0.664** |
| **High Phenotypic Burden (≥2)** | **4 (36.4%)** | **5 (55.6%)** | **-** | **0.262** |
| **B. Adult Patients (n=41)** | **n=5** | **n=36** | **Descriptive Analysis** |  |
| **Structural complexity** | **0 (0.0%)** | **4 (11.1%)** | **-** | **-** |
| **Aggressive features** | **5 (100.0%)** | **25 (69.4%)** | **-** | **-** |
| **Spatial complexity** | **3 (60.0%)** | **21 (58.3%)** | **-** | **-** |
| **High Phenotypic Burden (≥2)** | **3 (60.0%)** | **18 (50.0%)** | **-** | **-** |

Note: For Group A (pediatric), data are presented as number (percentage); between-group comparisons used Fisher's exact test. For Group B (adult), due to the small sample size of Streptococcus infections (n=5), statistical testing was not performed, and results are presented for descriptive analysis only.

**Supplementary Table 3. Profile of Imaging Phenotypes and Outcomes Across Different Pathogen Categories**

| **Pathogen Category** | **Cases (n)** | **Structural Complexity** | **Aggressive Features** | **Spatial Complexity** | **High Phenotypic Burden (≥2)** | **Adverse Outcome** |
| --- | --- | --- | --- | --- | --- | --- |
| **Streptococcus spp.** | **16** | **3 (18.8%)** | **13 (81.3%)** | **5 (31.3%)** | **6 (37.5%)** | **3 (18.8%)** |
| **Staphylococcus spp.** | **6** | **2 (33.3%)** | **4 (66.7%)** | **2 (33.3%)** | **2 (33.3%)** | **1 (16.7%)** |
| **Gram-negative bacilli** | **20** | **3 (15.0%)** | **10 (50.0%)** | **8 (40.0%)** | **7 (35.0%)** | **3 (23.1%)** |
| **Polymicrobial mixed infection** | **11** | **4 (36.4%)** | **8 (72.7%)** | **6 (54.5%)** | **6 (54.5%)** | **2 (18.2%)** |
| **Pathogen unspecified** | **23** | **0 (0.0%)** | **14 (60.9%)** | **6 (26.1%)** | **8 (34.8%)** | **2 (8.7%)** |

Note: This table presents an exploratory description based on cases with definitive microbiological results, aiming to show the distribution patterns of phenotypes and outcomes across different pathogen categories. Formal statistical comparisons between groups were not performed due to limited sample sizes in subgroups. Percentages are calculated based on the 'Cases (n)' for each row. Adverse outcome rate calculated from 13 cases with available outcome data in this subgroup.

**Supplementary Table 4. Common Co-occurrence Patterns of Imaging Phenotypes in Patients with High Phenotypic Burden**

| **Phenotype Combination** | **Overall (n=30)** | **Proportion of High-Burden Patients** | **Number with Adverse Outcome** |
| --- | --- | --- | --- |
| **Structural complexity + Aggressive features** | **10** | **33.3%** | **2** |
| **Aggressive features +**  **Spatial complexity** | **14** | **46.7%** | **3** |
| **All three phenotypes** | **6** | **20.0%** | **4** |

Note: This table is a descriptive analysis based on the 30 patients with high phenotypic burden (≥2 phenotypes). Statistical comparisons between co-occurrence patterns and outcomes were not performed due to sample size limitations.
